# fcGENE: A Versatile Tool for Processing and Transforming SNP Datasets

**DOI:** 10.1371/journal.pone.0097589

**Published:** 2014-07-22

**Authors:** Nab Raj Roshyara, Markus Scholz

**Affiliations:** 1 Medical Department, Institute for Medical Informatics, Statistics and Epidemiology, University of Leipzig, Leipzig, Germany; 2 Medical Department, LIFE Research Center (Leipzig Interdisciplinary Research Cluster of Genetic Factors, Phenotypes and Environment), University of Leipzig, Leipzig, Germany; University of Granada - Q1818002F, Spain

## Abstract

**Background:**

Modern analysis of high-dimensional SNP data requires a number of biometrical and statistical methods such as pre-processing, analysis of population structure, association analysis and genotype imputation. Software used for these purposes often rely on specific and incompatible input and output data formats. Therefore extensive data management including multiple format conversions is necessary during analyses.

**Methods:**

In order to support fast and efficient management and bio-statistical quality control of high-dimensional SNP data, we developed the publically available software fcGENE using C++ object-oriented programming language. This software simplifies and automates the use of different existing analysis packages, especially during the workflow of genotype imputations and corresponding analyses.

**Results:**

fcGENE transforms SNP data and imputation results into different formats required for a large variety of analysis packages such as PLINK, SNPTEST, HAPLOVIEW, EIGENSOFT, GenABEL and tools used for genotype imputation such as MaCH, IMPUTE, BEAGLE and others. Data Management tasks like merging, splitting, extracting SNP and pedigree information can be performed. fcGENE also supports a number of bio-statistical quality control processes and quality based filtering processes at SNP- and sample-wise level. The tool also generates templates of commands required to run specific software packages, especially those required for genotype imputation. We demonstrate the functionality of fcGENE by example workflows of SNP data analyses and provide a comprehensive manual of commands, options and applications.

**Conclusions:**

We have developed a user-friendly open-source software fcGENE, which comprehensively supports SNP data management, quality control and analysis workflows. Download statistics and corresponding feedbacks indicate that software is highly recognised and extensively applied by the scientific community.

## Introduction

Modern developments in micro-array techniques enable large scale genome-wide association (GWA) studies comprising thousands or millions of SNPs in thousands of individuals. Statistical methods for analysing GWA data were further developed in the last decade to handle several issues of GWA analysis such as principal component analysis (PCA), genotype imputation, haplotype-based analyses and different types of association models. A variety of software packages and environments have been developed to allow corresponding computations even for high-dimensional data. However, these software packages usually require their own specific input and output formats of data. As such, there are not only computational and statistical challenges in GWA analysis, but also a burden of fast management of different data formats and their transformations. Some apparent issues of necessary data transformations required in GWA analyses are described in the following.

PLINK [1], which is now the most popular and computationally efficient software for a variety of GWA analyses, requires a “ped”-file format containing a genotype matrix (one row per individual and one column per SNP) and a “map”-file that contains information on SNPs (one row per SNP). Software EIGENSOFT [2] is designed to perform PCA for example to analyse ethnical structures in genetic data. Even though the file format used by EIGENSOFT is very similar to PLINK-formatted “ped”- and “map”-files, some minor adaptations of the data format are required. One example is that EIGENSOFT uses “−99” to code missing phenotype information while PLINK uses “−9” as default for missings. Haploview, which is also an open source program, performs linkage disequilibrium (LD) analysis and estimates haplotype population frequencies [3]. This software basically requires two files: (a) a pedigree genotype data, and (b) a SNP annotation file with columns of marker names and base pair positions. PLINK-formatted pedigree data (“ped”) are accepted by Haploview, however it still requires an extra file with marker names and position information. Thus applying previously mentioned GWA software is possible only after a data conversion process, especially when genotype data are given in formats other than PLINK.

Genotype imputation has now become a standard process, especially in the case of meta-analyses combining data genotyped at different micro-array platforms [4]. Here, tasks related to data management are of particular concern. The data managing tasks comprise merging differently formatted multiple genotype data with different SNP content and individuals, converting the merged data into a format required by the selected imputation tool, and finally converting the imputation results back into different formats for down-stream analysis. A variety of tools such as MACH [5], IMPUTE [6]–[8], BEAGLE [9], BIMBAM [10], [11], PHASE [12] and fastPHASE [13] are available for genotype imputation. All these packages have different input and output formats. Moreover, there are in general many output formats of imputation results available such as most likely genotypes or genotype probability distribution for each SNP and individual. Some imputation software such as MaCH require a matrix row to describe an individual's genotypes while others (such as IMPUTE) require one row per SNP. In addition, the software may use one, two or three cells (e.g. “A/B”, “A B”, “0 1 0”) of a genotype matrix to describe a particular genotype. To allow imputation within an acceptable time frame, new strategies suggest chunking of chromosomes into small segments with a certain overlap which can be imputed by serial computations. Applying such strategies requires additional data management namely division of the main data set into small overlapping chunks and merging the overlapping imputation results.

Different strategies are proposed to test for association between imputed genotypes and different traits of interest. The most popular strategies are based on the following three types of imputed genotype data [14]: (a) best guess of genotypes (maximum a posteriori genotype), (b) expected minor allelic dosage, and (c) posterior-probabilities of the three possible genotypes. PLINK can deal with all three genotype representations but has little support for analyses addressing expected minor-allele-doses and individual genotype probabilities. SNPTEST [6]–[8] offers a Bayesian test for the analysis of single SNP association in GWA studies. The main input file of SNPTEST is similar to the output of IMPUTE, but another burden here is to create the required sample covariate file. GenABEL [15] is a package of the statistical software environment R [16]. It is another popular program for easy and fast analysis of genetic data. It uses a two bit format to efficiently store genotype data. Moreover, this package features R-functions to integrate IMPUTE-imputed and MaCH-imputed results but has no interface to convert BEAGLE-imputed and BIMBAM-imputed results.

Summarizing these issues, in state-of-the-art GWAS analyses we have to deal with differently formatted sets/subsets of high-dimensional genotype data. So far there is little support in generating or converting these different sets/subsets of genotype data. The usual way in dealing with this challenge is the application of self-written Linux based shell, Perl or R scripts. However such types of private solutions are cumbersome and prone to errors. Additionally, computer programming skill is necessary to write efficient format-converting scripts. Therefore, there is an obvious need for a user friendly genotype format converting and data management tool, which can handle the two-way format-conversions of genotype data in one framework. In this paper, we present and demonstrate the newly developed open-source tool fcGENE (format converting tool for genotype SNP data). This tool includes a variety of functions for the different genotype format conversions sketched previously and other useful options to support the process of SNP data management.

## Methods

### Basic Concepts

We developed fcGENE using C++ object-oriented programming language. Therefore it allows us to handle high-dimensional data sets quickly. Our aim was to construct fcGENE as a complementary tool to PLINK by developing options for transforming SNP data into the formats required by different tools for GWA analysis. Therefore interface and commands of fcGENE are inspired by PLINK commands. For example just like in PLINK, PLINK-formatted files “example.ped” and “example.map” can be read in fcGENE with the Linux based command option: “./fcgene --ped example.ped --map example.map” or “./fcgene --file example”. The sequential order of the command options is unimportant throughout. Each option contains a command identifier. Commands are separated by “--”. fcGENE runs under Unix, Linux and Microsoft Windows operating systems; the latter by either implementing it in MINGW [17] or in the statistical software R [16]. For example, PLINK-formatted binary files can be read in R as follows:

system (“./fcgene --bim example.bim --fam example.fam --bed example.bed”).

### Software validation

We have extensively checked and validated the correctness of the commands used in fcGENE. All functions related to format conversions were validated by performing multiple closed loops of format conversions, i.e. we converted genotype data from format “A” to format “B”, format “B” to format “C”, and finally, format “C” back to format “A”. After such a closed loop, we used Linux/Unix commands namely “diff” and “sdiff” to check if the first file containing original data in format “A” is identical to the result of the multiple conversions. Calculations of quality parameters (call rates, minor allele frequency (MAF), p-values of Hardy-Weinberg disequilibrium) were checked by comparing our results with those of PLINK. Many functions of fcGENE were also checked by repeating the same tasks via self-written Linux based shell-scripts or R scripts.

### Limitations

Functionality of fcGENE is based on the file formats required by current versions of GWA analysis tools. If future versions of any of these GWA tools bring some changes in their file formats, an update of fcGENE may be necessary to handle the new data formats properly. Current version of fcGENE is not designed for purposes of statistical computations and data analyses except for a few summary statistics and issues of quality control necessary for data management.

## Results

### Functional overview

**Figure 1:**
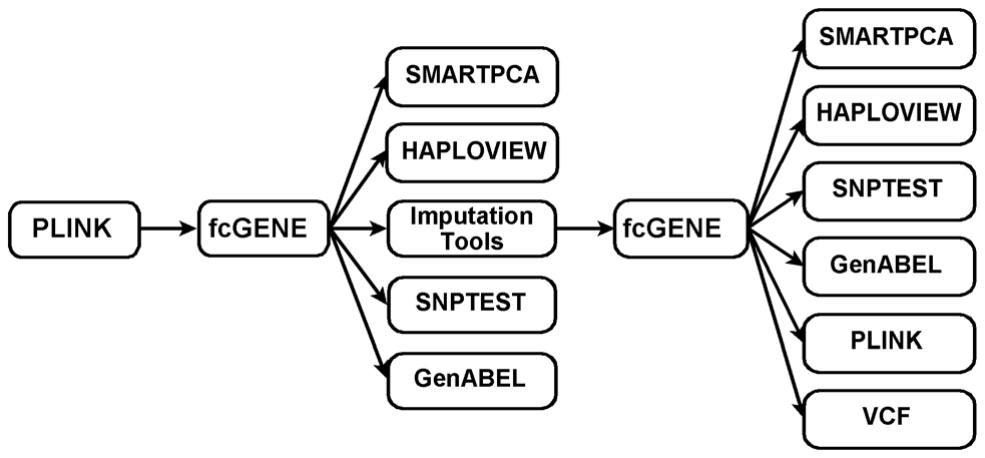
Flowchart of possible conversion steps for genotype data and the use of fcGENE during this process. Arrows pointing towards the fcGENE box imply that the data files of the programmes from where the arrows start can be read and uploaded by fcGENE. Similarly, arrows pointing away from fcGENE address programmes whose files can be generated.

fcGENE converts genotype data into different formats by loading inputs or outputs of a large variety of software packages such as PLINK, MaCH, IMPUTE, BEAGLE, BIMBAM, PHASE, fastPHASE and SNPTEST. It can also read and write compressed files (ending with “.gz”). If the original data is given in PLINK-format, we can use fcGENE to convert the data into the formats of either any imputation tool or that of other tools useful for genetic data analysis, such as SNPTEST, EIGENSOFT, HAPLOVIEW, GenABEL or VCF-tools. After completion of genotype imputation, fcGENE can be used to transform the imputation outputs back into the formats required for further analysis. A flowchart of possible data conversions is shown in Figure 1. In Figure 1, arrows pointing towards the fcGENE box imply that the data files of the programs from where the arrows start can be read and uploaded by fcGENE. Similarly, arrows pointing away from fcGENE address programs whose files can be generated. fcGENE can convert not only the inputs and outputs of imputation software, but also the imputation reference panels. This type of format conversion is required for example if we want to compare study genotypes and imputation reference panels.

Aside from the format converting tasks, fcGENE offers a number of additional options related to data management processes such as filtering SNPs and individuals according to pre-defined cut-offs of quality measures, generating templates of commands to run genotype imputation software, preparing phenotype files for SNPTEST software and GenABEL, and, creating scripts for EIGENSOFT and GenABEL.

### Syntax and command options

fcGENE uses unique command identifiers to recognize command options and the names of files to be loaded. For example command identifiers “--ped” and “--dat” recognize genotype data given in MERLIN (MaCH) format. Commands of fcGENE are inspired by PLINK's command-line syntax. This makes fcGENE commands intuitive for PLINK users. The names of command options also hint to their functions. For example, if genotype SNP data is given in PLINK format, we can use the following commands to prepare inputs of EIGENSOFT and HAPLOVIEW respectively.

./fcgene --ped plink.ped --map plink.map --oformat eigensoft --out plink_eigensoft

./fcgene --ped plink.ped --map plink.map --oformat haploview --out plink_haploview

Here, the command “--oformat” performs translation of data into the specified formats, e.g. those of EIGENSOFT and HAPLOVIEW respectively. Details are explained in the next section. Names of output files generated by fcGENE can be specified by using option: “--out”. More examples of format conversions can be found in the supplementary document. Command options required to upload data in fcGENE are listed in Table S1.

### Main functions

Two most important functions of fcGENE are format conversion of raw genotype data and transformation of imputed data into the formats required for different GWA tools. fcGENE can convert sets of genotype data of different formats into the formats of any of the specified imputation tools. Similarly, the outputs of different imputation software can be converted back either into PLINK format or into the formats of other software. Table 1 summarizes corresponding important command options implemented for fcGENE.

**Table 1:** Major command options of fcGENE.

Command options	Description
--ped, --map (or just --file)	reads plink-formatted ped and map file.
--bim --fam --bed (or just --bfile)	read plink-formatted binary file
--dosage, --fam	read plink-formatted dose file, fam file
--recodeA, --recodeAD	read plink-formatted raw files
--covar, --covar-name,--covar-type	read plink-formatted covariate file for create SNPTEST's sample file
--ped --dat (or just --mfile)	read Merlin (MaCH) formatted ped and dat files
--mach-hap, --mach-snp	read MaCH-formatted references file
--mach-geno, mach-info--mach-mlgeno, --mach-mlprob,	read MaCH-imputed files.
--gens	read IMPUTE-formatted input and output files
--impute-hap, impute-legend	read IMPUTE-formatted reference files
--haps, --sample	read SHAPEIT-phased HAPS and sample files
--bgl, --bgl-gprobs	read input and output of BEAGLE tools
--rgeno --snpinfo	read standard genotype data with counts of reference allele (0, 1, 2)
--wbg --pos --wgd	read to read input and output BIMBAM
--oformat	converts the uploaded data into the specified formats
--hardy, --crate, --freq	calculate SNP summary statistics: p-value of HWE, call rate, allele frequency
--filter-snp, --filter-indiv	-filter SNPs/individuals based on the cut-offs of quality-control measures
--snpinfo, --pedinfo	read files containing Phenotype and SNP information
--force	forces fcGENE to assign different phenotype information and SNP information to specified values or strings, e.g. to assign all individuals to males or females, case or controls etc.

We present the most relevant commands to read, manage and convert SNP data of different formats. Detailed information on each of the command options can be found in the supplementary document of this paper.

Imputation outputs are either most likely genotypes of SNPs for each individual or the genotype probability distribution for each SNP and individual. In addition, the outputs are either provided in rows per individual (e.g. MaCH- and MINIMAC-outputs) or one, two or three columns per individual (e.g. IMPUTE- and BEAGLE-outputs). fcGENE can convert all kinds of imputation outputs. After loading an original or imputed dataset into fcGENE, it can be converted into the format of any of the specified programs using the “--oformat” command. Further details about the command “--oformat” can be found in Table S2. Table S3 describes commands which are optional to use. These optional commands are used to update phenotype and SNP information, to calculate quality measures of SNPs and individuals, to apply corresponding filters and to split or merge genotype data. For example, MaCH-outputs do not contain SNP annotations such as base pair position. However, HAPLOVIEW requires base pair position to identify the genetic distance between two SNPs which can be loaded to fcGENE using command “--snpinfo”. Similarly, IMPUTE-outputs do not contain any kind of pedigree information. Therefore it is necessary to update the original phenotype information with “--pedinfo” option, before the impute outputs are converted into other data formats such as those required for PLINK. Files required to update SNP and pedigree information are automatically generated by fcGENE when data transformation is initiated. Alternatively, one can use “--write-snpinfo” and “--write-pedinfo” commands to generate SNP and pedigree information respectively. Detailed information on different command options can be found in the supplementary file and fcGENE's documentation, which is distributed together with the source code through its open source home page [18].

To analyse genotype - phenotype associations using allele dosages, one can convert the imputation output for example into PLINK's dosage-file-format using command option “--oformat plink-dosage”. Moreover, imputed data can be converted into other tools like HAPLOVIEW, EIGENSOFT and SNPTEST as well. Another output option for imputed data is to create a file containing dosages of minor alleles calculated on the basis of genotype probability distributions. This is achieved by the command “--oformat recodeA-dose”. The most recent version of BEAGLE (BEAGLE4) requires VCF-format [19]. The current version of fcGENE (1.0.7) can export VCF-formatted files using the option “--oformat vcf” (see Table S2). A read-option for VCF-formatted data will be added in a future version of the software.

### Auxiliary functions

For the convenient application of fcGENE, we implemented a number of additional options and features, namely options related to execution of multiple commands at a time, data management like merging, splitting, exclusion of SNPs and individuals, quality control of both raw and imputed genotypes, generation of templates for software commands and updating phenotype and SNP information. Some of these functions are described below.

### Execution of multiple tasks

fcGENE can execute multiple tasks, i.e. fcGENE can process two or more tasks by one command. Each new task, except the first, starts with identifier “--new-start” and ends with “--new-end”. This command can be used for example to merge two or more sets of genotype data. The following example command reads two different PLINK-formatted files and convert the first one into MaCH and second one into IMPUTE format.

.*/fcgene --gens example1_impute.gens --oformat mach*

*--new-start --dosage example2.dose --fam example2.fam --map example2.map\*

*--oformat impute --out impute/example2 --new-end --out mach/example1*

If option “--merge” is also used (see supplementary document) within “--new-start” and “--new-end” then the genotype data processed within these two identifiers, are merged with the genotype data loaded first (i.e. command given outside of the “--new-start” and “ --new-end” environment). Examples of merging data are given in the supplementary document.

### Strand alignment

Strand alignment between genotype data set and reference data set is crucial for GWA analysis and imputation. Generally, reference panels such as HapMap are given as ‘+’ strand but data might be genotyped with respect to negative strand. If two samples at a SNP are genotyped at different strands, it can be easily recognized except for C/G or A/T SNPs. PLINK has the option to detect opposite strand alignments between cases and controls (“--flip-scan”). fcGENE supports the comparison of strand information between genotyped SNP data and reference panels using this PLINK's “--flip-scan” feature in the following way: (1) Use fcGENE to merge study genotypes and the corresponding reference panel, (2) use fcGENE to convert the merged data into PLINK format and assign a dummy case (genotyped data) and control (reference) status using option “--force”, (3) use PLINK to detect the strand mismatches of ambiguous markers applying command option “--flip-scan” or “--flip-scan-verbose” [1] on the merged data, (4) create a list of SNPs whose strand needs to be flipped, (5) use PLINK to flip the strand and (6) use fcGENE finally to convert the corrected genotypes into the format required by the desired imputation tool.

### Pre-imputation quality control

It is common to implement a series of quality control (QC) steps at SNP-wise and sample-wise level before and after genotype imputation so that different confounding factors, which might affect imputation quality, can be ruled out. Typical SNP-wise QC measures comprise p-value of HWE-test, MAF and genotyping call rate [20]. Similarly, sample-wise genotyping call rate is also often applied as filter criterion. In the command line of fcGENE, one can prescribe the thresholds of these SNP-wise and sample-wise quality measures so that SNPs or individuals violating these criteria are automatically discarded. While filtering SNPs and samples, the default process of fcGENE calculates all quality measures without excluding any SNPs and samples of the uploaded data, and then filters SNPs and samples according to the specified thresholds. SNP-wise and individual-wise thresholds can be assigned by the command options “--filter-snp” and “--filter-indiv” respectively.

### Generating templates of software commands

fcGENE can create not only files required by specified software, but it also generates scripts required to run the software. For example if we use IMPUTE, we have to provide lower and upper limits of the base pair positions to be imputed. Moreover, if we analyze a whole chromosome with IMPUTE, we may have to split it into smaller chunks to parallelize computations. Since it is cumbersome to specify the upper and lower limits of the chunks manually, fcGENE generates both, a list of upper and lower limits of base pair positions for each chunk, a Perl script that can execute all commands of the chunking process, and, a list of commands used to impute the chunks as well.

To start the imputation process, we may require a set of imputation reference panels. Imputation commands only work properly if one provides filenames of the reference panel with correct file identifiers. To support this process, fcGENE generates appropriate templates which can be edited with respect to filenames and folders of the reference panel.

While converting files into EIGENSOFT format, fcGENE also generates parameter files and command templates necessary for running SMARTPCA and SMARTEIGENSTRAT. More precisely the extra files generated by fcGENE are: a parameter file for SMARTPCA, a parameter file for SMARTEIGENSTRAT, an R-script for generating PCA-Plots and for modifying outputs of SMARTPCA to be compatible with the format of SMARTEIGENSTRAT, and finally, a Linux-script (alternative way of using R) to run SMARTPCA, SMARTEIGENSTRAT, EIGENPLOT and TWSTATS. More information on the parameter files and the different packages of EIGENSOFT can be found on its official website [21].

### Post-imputation quality control

fcGENE can filter poorly imputed SNPs on the basis of their imputation quality and allele frequency. Imputation quality metrics differ between imputation tools. For example, MaCH uses quality parameter “Rsq” to assess imputation quality, while IMPUTE calculates so called “quality” and “info” scores (see corresponding publications for more information regarding definition of these QC parameters). The choice of corresponding filter options is rather intuitive: For example, filtering SNPs with values of MaCH-imputation quality score (i.e. Rsq score) lower than 0.3, is achieved by “--rsq 0.3”. Similarly, filtering SNPs with allele frequency <1% is achieved by the option “--maf-thresh 0.01”. An overview of possible options regarding post-imputation quality control can be found in Table S3. Detailed information on the filtering process are given in the manual of fcGENE [18].

### Updates of SNP and sample information

To create a list of SNPs and individual ids from the uploaded genotype data, fcGENE provides command options “--write-snplist” and “--write-pedlist” respectively. Not all software need whole information related to SNPs and samples contained in genotype data. Therefore it may be necessary to drop or add SNP and sample information before one converts genotype data between formats. Moreover, it may be necessary to define coding and non-coding allele before reading and converting raw genotype data and probability distributions of imputed genotypes. fcGENE provides command options “--snpinfo” and “--pedinfo” respectively to add or update SNP information like rsid, allele information, base pair position etc, and pedigree information like pedigree ids, phenotype status and sex information.

Identifying individuals in pedigree data format requires family, parental and individual ids. However, some software like SHAPEIT or EIGENSOFT accept only one ID for each individual. In such a case, fcGENE can create hybrid ids by combining family-ids, individual-ids, paternal ids or maternal ids using the command option “--iid”. The process for creating such hybrid sample ids is explained in Table S4 and in fcGENE's documentation file in more detail.

While converting data for SNPTEST, we may require covariate information. Option “--covar” reads files given in plink-formatted covariate file and updates necessary information prior to data transformation.

### Adding group labels in preparation for PCA analysis

To plot the result of PCA, software SMARTPCA expects an assignment of each sample to its population/ethnic group. To add this group labelling, fcGENE accepts an extra file (containing sample ids in the first column and group/population-labelling of the samples in the second column) using command option “--group-label”.

### Coding genotypes as count of a given allele

fcGENE can code genotypes as counts of a given allelic reference. This type of coding is convenient for a variety of different statistical analyses such as regression models [14]. PLINK supports generation of files containing counts of minor alleles. However it only supports data transformation from the raw calls of genotype data resulting in numbers 0, 1 and 2 of the minor allele dose. Complementary to this option, fcGENE facilitates transformation of genotype probability distributions into PLINK's recodeA-formatted files but using expected allele doses of a given reference allele. More precisely, the raw files transformed by fcGENE can contain not only 0, 1 and 2 as reference allele counts but also expected allele doses of the reference allele, i.e. numbers between 0 and 2. The default reference allele is the minor allele. However, users can force fcGENE to take either the first, or the second or the major allele as reference. GWA analysis with this type of coding is useful especially if the uncertainty of imputation results is high [14].

To facilitate analyses of imputed genotype data with the statistical package R, fcGENE can convert sets of genotype data from different formats into standard text files with genotype codes either as counts of a reference allele (0,1 or 2), or as the expected dose of the reference allele. Text files contain rows for samples and columns for SNPs with simple headers for SNP identifier (e.g. rs-IDs) and a first column to identify subjects (i.e. sample ids). In order to write SNPs as rows and individuals as columns, an additional command option “--transpose” can be used. Command options “--oformat r” and “--oformat r-dose” are used to write the allele counts and expected doses of the reference alleles. Again, the default reference allele is the minor allele. However one can alter the reference allele using commands like “--force ref-allele = major”. Re-import of these types of allele counting data is also possible (command option “--rgeno”). More information on these formats can be found in the supplementary file.

### An example workflow

The following example workflow demonstrates how fcGENE can be applied in different stages of GWA analysis, and how it interacts with PLINK. We assume that original genotype data are in PLINK format and saved as “example.ped” and “example.map” files. If we plan to create a plot for PCA analysis after quality control, one can use the following command to convert the data into EIGENSOFT format.

.*/fcgene --ped example.ped --filter-snp hwe = 1e-6,crate = 0.95,maf = 0.10\*

*--map example.map --oformat eigensoft --out example_eigensoft*

This command filters SNPs with low quality and low minor allele frequencies, saves files and writes scripts necessary to run software EIGENSOFT at different stages. In addition, the command also creates R-scripts to plot PCA results on the basis of outputs of SMARTPCA.

In the next step, we aim to impute the PLINK-formatted data with IMPUTE using HapMap reference panel and to analyse the imputation output with SNPTEST after performing post-imputation quality control. We start this work by comparing the strand similarities between study genotypes and IMPUTE-formatted reference panels as explained in section “strand alignment”. A typical command line for this process is given below.

..*/fcgene --ped example.ped --example.map --force pheno = aff\*

*--new-start --impute-hap impute_ref.hap --impute-legend impute_ref.legend\*

*--merge --force pheno = unaff --new-end --oformat plink --out merged_data*

After correcting the mismatched strand with PLINK, we can convert the study genotypes into IMPUTE format applying pre-imputation quality control as follows.

.*/fcgene --ped example.ped --map example.map\*

*--filter-snp hwe = 1e-6,crate = 0.95,maf = 0.01 --filter-indiv crate = 0.95\*

*--oformat impute --out example_impute*

This command also generates the files necessary to run IMPUTE. After finishing genotype imputation, we can convert the results into SNPTEST format applying post-imputation quality filtering.

.*/fcgene --gens example_impute2 --thresh 0.9\*

*--info example _info --info-thresh 0.3 --filter-snp --maf-thresh 0.1\*

*--pedinfo example/impute.pedinfo --out example_snptest*

### Access statistics

fcGENE is widely used by several research institutions. We received a number of positive feedbacks of active users. Since the first release of fcGENE on 2012-09-26, the software has been downloaded more than 2000 times from more than 60 countries. The highest number of downloaders are from USA (N = 599), followed by UK (N = 214), Germany (N = 138) China (N = 131), and Spain (N = 70). These statistics were taken from fcGENE's official sourceforge website: http://sourceforge.net/projects/fcgene/ on 2014/03/12.

## Discussion

Different analysis tools having their own specific input- and output formats are in use in modern GWAS analysis. Motivated by recurrent format conversions during GWAS analysis processes, we developed the open source format converting tool fcGENE. This software automates the process of transformation of genotype data among the most common formats required by different GWA tools with emphasis on imputation software. We also provide a number of helpful features facilitating the data management process of comprehensive data analysis pipelines such as quality control on the basis of usual measures of genotype and sample quality, splitting and merging of genotype data sets, exclusion of SNPs and individuals, updating SNP annotation and sample information, and, generation of command templates necessary to execute specific tools. Rather than constructing another self-contained software for statistical analyses, we developed fcGENE in order to make the use of existing GWA packages easier. Through this, we simplify and automate the process of imputation-based GWA studies. The tool has been developed on the basis of C++ which allows dealing with large datasets quickly. Syntaxes of fcGENE are similar to those of PLINK. Therefore PLINK users may find fcGENE easy and intuitive to apply. fcGENE has gained many regular user world-wide and a number of positive feedbacks encouraged us to further improve and develop the software.

The current version of the software is able to perform data format conversion between the analysis packages EIGENSOFT, HAPLOVIEW, PLINK, R (GENABEL package), SNPTEST and the imputation packages BEAGLE, BIMBAM, IMPUTE, MACH, PHASE, fastPHASE and PLINK. Conversions involving the phasing software SHAPEIT [22] can also be addressed since it accepts PLINK or IMPUTE formatted data as input and outputs IMPUTE formatted data. Moreover, fcGENE can convert SHAPEIT-formatted phased data (*.haps and *.sample) file into other formats.

There are only a few publically available tools for the purpose of data transformation: GTOOL [23] is one of such programs and is provided by the IMPUTE developers. This program solely supports transformations between PLINK-formatted ped/map files and IMPUTE-formatted gen/sample files. Similarly, MaCH software developers provided some templates of Perl scripts, which can deal with data transformation of MaCH-imputed data. However, as mentioned previously, without efficient computer programming knowledge, such template scripts are difficult to edit. GenGen is another Perl program [24] for genotype format conversions. However, this program supports only conversions of MaCH-imputed data into PLINK-formatted ped/map files and into the file formats required by SNPTEST. Aside from these tools, one may find some private R-scripts or Perl-scripts for selected software-specific format conversion at different websites. Hence, to our knowledge there is no software with comprehensiveness comparable to that of fcGENE.

### Future plans for software extensions

The recent version of fcGENE allows export of genotype data into the variant call format (VCF). In the next step, an option to import this data format will be added. We plan to develop fcGENE as format converter of family data and to add a graphical user interface (GUI) for windows users. We are also committed to update fcGENE if necessary, especially in case of changes of input and output formats of the addressed software packages.

## Supporting Information

Table S1 **Commands to read SNP data of different formats.** Table S1 summarizes command options necessary to upload genotype data of different formats into fcGENE. In the table, we used the name “example” as file name combined with different extensions specific for different data formats.
(DOCX)

Table S2 **Commands to generate file formats required for different GWA analysis tools.**
(DOCX)

Table S3 **Optional commands used in fcGENE.** These options comprise specification and application of quality cut-offs as well as provision of supplementary data. A description of each of the commands is given in the second column.
(DOCX)

Table S4 **Command options to create new IDs on the basis of pedigree information.**
(DOCX)

Text S1 **Command Summary.**
(DOCX)

## References

[1] Purcell S, Neale B, Todd-Brown K, Thomas L, Ferreira MAR, et al (2007) PLINK: a tool set for whole-genome association and population-based linkage analyses. Am J Hum Genet 81: 559–575 10.1086/519795 17701901 PMC1950838

[2] Patterson N, Price AL, Reich D (2006) Population Structure and Eigenanalysis. PLoS Genet 2: e190 10.1371/journal.pgen.0020190 17194218 PMC1713260

[3] Barrett JC, Fry B, Maller J, Daly MJ (2005) Haploview: analysis and visualization of LD and haplotype maps. Bioinforma Oxf Engl 21: 263–265 10.1093/bioinformatics/bth457 15297300

[4] Marchini J, Howie B (2010) Genotype imputation for genome-wide association studies. Nat Rev Genet 11: 499–511 10.1038/nrg2796 20517342

[5] Abecasis GR (n.d.) Homepage of Imputation software MaCH1.0. Available: http://www.sph.umich.edu/csg/abecasis/MACH/tour/imputation.html.

[6] Marchini J (n.d.) Homepage of IMPUTE2. Available: https://mathgen.stats.ox.ac.uk/impute/impute_v2.html.

[7] Marchini J, Howie B, Myers S, McVean G, Donnelly P (2007) A new multipoint method for genome-wide association studies by imputation of genotypes. Nat Genet 39: 906–913 10.1038/ng2088 17572673

[8] Burton PR, Clayton DG, Cardon LR, Craddock N, Deloukas P, et al (2007) Genome-wide association study of 14,000 cases of seven common diseases and 3,000 shared controls. Nature 447: 661–678 10.1038/nature05911 17554300 PMC2719288

[9] Browning SR, Browning BL (2007) Rapid and accurate haplotype phasing and missing-data inference for whole-genome association studies by use of localized haplotype clustering. Am J Hum Genet 81: 1084–1097 10.1086/521987 17924348 PMC2265661

[10] Guan Y, Stephens M (2008) Practical Issues in Imputation-Based Association Mapping. PLoS Genet 4: e1000279 10.1371/journal.pgen.1000279 19057666 PMC2585794

[11] Servin B, Stephens M (2007) Imputation-Based Analysis of Association Studies: Candidate Regions and Quantitative Traits. PLoS Genet 3: e114 10.1371/journal.pgen.0030114 17676998 PMC1934390

[12] Stephens M, Smith NJ, Donnelly P (2001) A New Statistical Method for Haplotype Reconstruction from Population Data. Am J Hum Genet 68: 978–989 10.1086/319501 11254454 PMC1275651

[13] Scheet P, Stephens M (2006) A fast and flexible statistical model for large-scale population genotype data: applications to inferring missing genotypes and haplotypic phase. Am J Hum Genet 78: 629–644 10.1086/502802 16532393 PMC1424677

[14] Zheng J, Li Y, Abecasis GR, Scheet P (2011) A comparison of approaches to account for uncertainty in analysis of imputed genotypes. Genet Epidemiol 35: 102–110 10.1002/gepi.20552 21254217 PMC3143715

[15] Aulchenko YS, Ripke S, Isaacs A, van Duijn CM (2007) GenABEL: an R library for genome-wide association analysis. Bioinforma Oxf Engl 23: 1294–1296 10.1093/bioinformatics/btm108 17384015

[16] Chambers J, Bates D, Dalgaard P, Falcon S, Gentleman R (n.d.) R: A Language and Environment for Statistical Computing.

[17] MIGW: A minimalist GNU for Windows (n.d.). Available: http://www.mingw.org/.

[18] Roshyara NR, Scholz M (n.d.) fcGENE: Format converting toolset for genotyped data. Available: https://sourceforge.net/projects/fcgene/.

[19] Danecek P, Auton A, Abecasis G, Albers CA, Banks E, et al (2011) The variant call format and VCFtools. Bioinforma Oxf Engl 27: 2156–2158 10.1093/bioinformatics/btr330 PMC3137218 21653522

[20] De Bakker PIW, Ferreira MAR, Jia X, Neale BM, Raychaudhuri S, et al (2008) Practical aspects of imputation-driven meta-analysis of genome-wide association studies. Hum Mol Genet 17: R122–R128 10.1093/hmg/ddn288 18852200 PMC2782358

[21] Patterson N, Price AL, Reich D (2006) Population Structure and Eigenanalysis. PLoS Genet 2: e190 10.1371/journal.pgen.0020190 17194218 PMC1713260

[22] Delaneau O, Marchini J, Zagury J-F (2012) A linear complexity phasing method for thousands of genomes. Nat Methods 9: 179–181 10.1038/nmeth.1785 22138821

[23] Colin F (n.d.) Homepage of Genotype Format converting Tool: GTOOL. Available: http://www.well.ox.ac.uk/~cfreeman/software/gwas/gtool.html.

[24] Wang K, Li M, Bucan M (2007) Pathway-based approaches for analysis of genomewide association studies. Am J Hum Genet 81: 1278–1283 10.1086/522374 17966091 PMC2276352

