# Supplementary material for: fcGENE: A Versatile Tool for Processing and Transforming SNP Datasets
Source: PLoS One. 2014 Jul 22;9(7):e97589. doi: 10.1371/journal.pone.0097589 (PMC4106754; doi:10.1371/journal.pone.0097589)
Supplement: Table S1 — Commands to read SNP data of different formats. Table S1 summarizes command options necessary to upload genotype data of different formats into fcGENE. In the table, we used the name “example” as file name combined with different extensions specific for different data formats. (DOCX) [file pone.0097589.s001.docx]

**Table S1:** **Commands to read SNP data of different formats.** Table 1 summarizes command options necessary to upload genotype data of different formats into fcGENE. In the table, we used the name “example” as file name combined with different extensions specific for different data formats.

| **Type of file format** | **Command options to load data into fcGENE** |
| --- | --- |
| PLINK | --ped example.ped --map example.map or --file example |
| PLINK-Binary | --bed example.bed --bim example.bim --fam example.fam or --bfile example |
| plink-formatted  covariate file | --covar examplecov.txt --covar-name pheno1,pheno2,covar_A,covar_B --covar-type P,B,D,C  (These options are necessary while preparing files for SNPTEST) |
| PLINK-formatted dosage file | -- dosage example.dosage --fam example.fam --map example.map |
| PLINK-raw (recodeAD type ) | --recodeADexample.raw –map example.map --snpinfo example_snpinfo.txt |
| PLINK-raw (recodeA type ) | --recodeA example.raw, --map example.map --snpinfo example_snpinfo.txt |
| MaCH | --ped example.ped -- dat example.dat or --mfile example |
| mach references | --mach-hap mach_ref.hap --mach-snp mach_ref.snp  --force pheno=unaff,sex=m (optional ) |
| mach imputation output | --mach-geno example.geno --mach-info example.info |
| mach imputation output | --mach-mlgeno example.mlgeno --mach-mlinfo example.mlinfo |
| mach imputation output | --mach-mlprob example.mlprob --mach-mlinfo example.mlinfo |
| minimac imputation output | --minimac-mlprob example.mlprob --minimac-mlinfo example.mlinfo |
| IMPUTE | --gens example.gens |
| impute references | --impute-hap impute_ref.hap --impute-legend impute_ref.legend  --force pheno=unaff,sex=m (optional) |
| impute imputation output | --gens example.impute2 --info example.impute2_info |
| BEAGLE | --bgl example.bgl |
| beagle references | --bgl beagle_ref.bgl  --force pheno=unaff,sex=m (optional) |
| beagle imputation output | --bgl example.bgl.phased --bgl-rsq example.bgl.r2 (optional) |
| beagle imputation output | --bgl-gprobs example.bgl.gropbs --bgl-rsq example.bgl.r2 (optional) |
| BIMBAM | --wbg example.geno.txt |
| bimbam imputation output | --wbg example.best.guess.genotype.txt  --pos example.snpdata.txt |
| bimbam imputation output | --wgd example. genotype.probability.distribution.txt  --pos example.snpinfo.txt |
| Standard genotype data with counts of reference allele(0, 1,2) | --rgeno genotype_data.txt --snpinfo allele_info.txt |
| SHAPEIT: *haps and *sample files | --haps example.haps --sample example.sample |
