# Supplementary material for: fcGENE: A Versatile Tool for Processing and Transforming SNP Datasets
Source: PLoS One. 2014 Jul 22;9(7):e97589. doi: 10.1371/journal.pone.0097589 (PMC4106754; doi:10.1371/journal.pone.0097589)
Supplement: Table S2 — Commands to generate file formats required for different GWA analysis tools. (DOCX) [file pone.0097589.s002.docx]

**Table S2: Commands to generate file formats required for different GWA analysis tools**

| **Type of program-files to be created** | **Command option** |
| --- | --- |
| PLINK (*.ped ,*.dat) | --oformat plink |
| PLINK binary (*.bim, *.bed, *.fam) | --oformat plink-bed |
| PLINK (dose file: *.dat and *.fam , *.map), | --oformat plink-dosage |
| PLINK (raw(recodeA type) file: *.raw and *.map), | --oformat plink-recodeA |
| PLINK-raw expected dose file: *.raw and *.map), | --oformat recodeA-dose |
| PLINK (raw(recodeAD type) file: *.raw and *.map), | --oformat plink-recodeAD |
| MaCH (*.ped , *.dat) | --oformat mach |
| minimac (*.snps, *.ped) | --oformat minimac |
| IMPUTE (*.gens, *.strand) | --oformat impute |
| BEAGLE (*.bgl) | --oformat beagle |
| BIMBAM (*.geno.txt, *.pos.txt) | --oformat bimbam |
| PHASE/fastPHASE | --oforamt phase /--oformat fastphase |
| SNPTEST (*.gens , *.sample ) | --oformat snptest |
| EIGENSOFT | --oformat eigensoft |
| HAPLOVIEW | --oformat haploview |
| R compatible files: *.geno.txt, *.affs.txt | --oformat R or --oformat r |
| Standard text file with expected doses of minor allele | --oformat r-dose or -- oformat R-dose |
| GenABEL | --oformat genable |
| VCF formatted files | --oformat vcf |
| Pedigree information (pedinfo.txt) | --write-pedinfo |
| SNP information (snpinfo.txt) | --write-snpinfo |
| A list of individuals (pedlist.txt) | --write-pedlist |
| A list of SNP/rsid (snplist.txt) | --write-snplist |
| writing a file with p-values of exact HWE test | --hardy |
| Calculation of SNP-wise and/or sample-wise callrate | --crate |
| Calculation of allele frequencies | --freq |
