# Supplementary material for: fcGENE: A Versatile Tool for Processing and Transforming SNP Datasets
Source: PLoS One. 2014 Jul 22;9(7):e97589. doi: 10.1371/journal.pone.0097589 (PMC4106754; doi:10.1371/journal.pone.0097589)
Supplement: Table S3 — Optional commands used in fcGENE. These options comprise specification and application of quality cut-offs as well as provision of supplementary data. A description of each of the commands is given in the second column. (DOCX) [file pone.0097589.s003.docx]

**Table S3: Optional commands used in fcGENE:** These options comprise specification and application of quality cut-offs as well as provision of supplementary data. A description of each of the commands is given in the second column.

| **Optional commands** | **Function of the command option** |
| --- | --- |
| --fitler-snp maf=0.1, crate=0.95, hwe=1e-2 | This command excludes SNPs that have smaller values of minor allele frequency, call rate, or p-value of Hardy-Weinberg equilibrium than the prescribed by the cutoff values defined by the commands maf=, crate= and hwe= respectively. Filters can be combined arbitrarily. |
| --filter-indiv crate=0.95 | Filters individuals with call rate lower than given cut-off value. |
| --pedinfo | By this command, one can provide an extra file containing pedigree information of individuals. |
| --snpinfo | This option is used to provide an extra file containing SNP annotations (e.g. rsid, base pair position). |
| --rsq 0.3 | This command can be used with MaCH- and MINIMAC-imputed data excluding SNPs with imputation quality (Rsq value) lower than given cutoff. Rsq is an estimation of the squared correlation between imputed and true genotypes |
| --thresh | While finding the best guess genotype for each individual at every SNP, one can use the threshold option --thresh 0.9 to guarantee that the best guess genotype has a probability equal or greater than 0.9. If the maximum probability of predicted genotypes is less than 0.9, then the genotype is set to missing. This option improves the confidence of best guess genotypes. |
| --rsq-thresh | This option is used to filter poorly imputed SNPs on the basis of squared correlation $R^{2}$used by BEAGLE as a measure imputation quality. |
| --info-thresh | This option is used for post-imputation filtering of IMPUTE-imputed data on the basis of the info score |
| --force | This option forces fcGENE to assign different phenotype information and SNP information to specified values or strings. e.g. --force option can assign to dummy sex status i.e. all individuals either to male or female |
| --ssplit n1-n2, n3-n4,... | This option extracts specified SNPs or can be used to split datasets SNP-wise. Numbers n1, n2, … refer to ranges of indices of SNPs to be extracted. Commas refer to the different data sets to be produced. |
| --bpsplit n1-n2, n3-n4, ... | This option also extracts specified SNPs or can be used to split datasets SNP-wise. Here, numbers n1, n2, … refer to ranges of base pair positions of SNPs to be extracted. Commas refer to the different data sets to be produced. This option is useful if all SNPs are from the same chromosome. |
| --isplit n1-n2, n3-n4, ... | This option extracts specified samples or can be used to split datasets sample-wise. Numbers n1, n2, … refer to ranges of indices of samples to be extracted. Commas refer to the different data sets to be produced. |
