# Supplementary material for: fcGENE: A Versatile Tool for Processing and Transforming SNP Datasets
Source: PLoS One. 2014 Jul 22;9(7):e97589. doi: 10.1371/journal.pone.0097589 (PMC4106754; doi:10.1371/journal.pone.0097589)
Supplement: Table S4 — Command options to create new IDs on the basis of pedigree information. (DOCX) [file pone.0097589.s004.docx]

**Table S4: Command options to create new IDs on the basis of pedigree information.**

| Command option | New IDs |
| --- | --- |
| - -iid famid,iid,sep=_ | famid_iid |
| - -iid famid,iid,patid,matid,sep=_ | famid_iid_patid_matid |
| - -iid famid,iid,patid,matid,sep=- | famid-iid-patid-matid |
| - -iid famid,iid,patid,matid | famidiidpatidmatid |
