# Supplementary material for: fcGENE: A Versatile Tool for Processing and Transforming SNP Datasets
Source: PLoS One. 2014 Jul 22;9(7):e97589. doi: 10.1371/journal.pone.0097589 (PMC4106754; doi:10.1371/journal.pone.0097589)
Supplement: Text S1 — Command Summary. (DOCX) [file pone.0097589.s005.docx]

**Text S1: Commands summary**

Here, we provide an overview of implemented command options of fcGENE.
**Example:** To upload BEAGLE output, one can use the command:

*./fcgene --bgl-gprobs example.bgl.gropbs*

After loading a dataset into fcGENE, it can be converted into the format of any of the programmes mentioned in Table S2. Data conversions are usually performed by combining an option of Table S1 with an option of Table S2. **Example:** If genotype SNP data are given in MaCH-imputed mlprob format, we can use the following command to prepare inputs of PLINK.

*./fcgene --mach-mlprob example.mlprob --mach-mlinfo example.mlinfo - -oformat plink*

In Table S3, we summarized different optional commands related to quality control and data manipulation. Users can adjust for example different threshold values for different quality measures such as imputation quality, minor allele frequency (MAF), p-value of Hardy-Weinberg equilibrium (HWE), call rate etc. and apply these filters to their data. The sequential order of options is unimportant throughout. The following command can be used to filter MERLIN (MaCH-input) formatted data regarding different quality issues:

*./fcgene --ped example.ped --dat example.map\*

*--filter-snp hwe=1e-2,maf=0.1,crate=0.95 --filter-indiv crate=0.95\*

*--oformat mach --out mach/example_qcd_data*

**File formats used by different GWA-tools:**

Genotype file formats used by different GWA tools are described in the following. We also describe how to load and create these formats with fcGENE.

**PLINK -format files: example.ped and example.dat**

- example.ped:

| fam_1 | ind_1 | 0 | 0 | 1 | 1 | T | T | T | T | G | G |
| --- | --- | --- | --- | --- | --- | --- | --- | --- | --- | --- | --- |
| fam_2 | ind_2 | 0 | 0 | 1 | 1 | G | G | C | T | G | A |
| fam_3 | ind_3 | 0 | 0 | 1 | 2 | T | G | T | T | A | G |
| fam_4 | ind_4 | 0 | 0 | 2 | 2 | G | G | C | C | 0 | 0 |

- example.map

| 22 | snp1 | 0.02 | 16212142 |
| --- | --- | --- | --- |
| 22 | snp2 | 0.13 | 20278224 |
| 22 | snp3 | 0.13 | 20304703 |

- fcGENE commands:
  - - To read files:

*./fcgene --ped example.ped --map example.map*

- - - To create this format:

*--oformat plink*

**PLINK -formatted dosage file: example.dosage, example.fam , example.map**

- example.dosage

| SNP | A1 | A2 | fam_1 | ind_1 | fam_2 | ind_2 | fam_3 | ind_3 | fam_4 | ind_4 |
| --- | --- | --- | --- | --- | --- | --- | --- | --- | --- | --- |
| snp1 | T | G | 0 | 1 | 0.05 | 0 | 0.02 | 0.97 | 0 | 0.03 |
| snp2 | C | T | 0.88 | 0.03 | 1 | 0 | 1 | 0 | 1 | 0 |
| snp3 | G | A | 0.78 | 0.2 | 1 | 0 | 0.91 | 0 | 1 | 0 |

- example.fam

| Fam_1 | ind_1 | 0 | 0 | 1 | 1 |
| --- | --- | --- | --- | --- | --- |
| Fam_2 | ind_2 | 0 | 0 | 1 | 1 |
| Fam_3 | ind_3 | 0 | 0 | 1 | 2 |
| Fam_4 | ind_4 | 0 | 0 | 2 | 2 |

- fcGENE commands:
  - To read files:

*./fcgene --dosage example.dose --fam example.fam\*

*--map example.map (optional)*

- - To create this format:

*--oformat plink-dosage*

**PLINK -formatted raw file (created with --recodeA in plink): example.raw, example_snpinfo.txt**

- example.raw :

| FID | IID | PAT | MAT | SEX | PHENOTYPE | snp1_T | snp2_C | snp3_A |
| --- | --- | --- | --- | --- | --- | --- | --- | --- |
| fam_1 | ind_1 | 0 | 0 | 1 | 1 | 2 | 0 | 0 |
| fam_2 | ind_2 | 0 | 0 | 1 | 1 | 0 | 1 | 1 |
| fam_3 | ind_3 | 0 | 0 | 1 | 2 | 1 | 0 | 1 |
| fam_4 | ind_4 | 0 | 0 | 2 | 2 | 0 | 2 | NA |

- example.map: see plink-formatted map file above.
- example_snpinfo.txt :

| nchr | snpid | rsid | bp | cm_pos | allele1 | allele2 |
| --- | --- | --- | --- | --- | --- | --- |
| 22 | snp1 | snp1 | 16212142 | 0.02 | T | G |
| 22 | snp2 | snp2 | 20278224 | 0.13 | T | C |
| 22 | snp3 | snp3 | 20304703 | 0.13 | G | A |

**Remark:** “example_snpinfo.txt” file can be loaded by fcGENE with option “--snpinfo”. Since plink-formatted raw file has no allele information, it is necessary to provide at least the allele information. Therefore, “example_snpinfo.txt “ must contain at least rsid, allele1 and allele2.

- fcGENE commands:
  - To read files:

*./fcgene --recodeA example.raw --map example.map\*

*--snpinfo example_snpinfo.txt\*

*--pedinfo example_pedinfo* (optional)

- - To create this format:

*--oformat plink-recodeA*

**PLINK-formatted raw dose file: example.raw**

This type of file format has the same form as previously mentioned PLINK raw files but provides expected allele doses of reference allele instead of genotypes resulting in numbers between 0 and 2. By default, minor-allele is taken as reference allele. One can force fcGENE to change the reference allele with command option “--force ref-allele=”. Possible options for forcing reference allele are “--force ref-allele=minor-allele” (this is default), “--force ref-allele=major-allele”, “--force ref-allele=allele1” and “--force ref-allele=allele2”.

- example.raw :

| FID | IID | PAT | MAT | SEX | PHENOTYPE | snp1_T | snp2_C | snp3_A |
| --- | --- | --- | --- | --- | --- | --- | --- | --- |
| fam_1 | ind_1 | 0 | 0 | 1 | 1 | 1.89 | 0.19 | 0.18 |
| fam_2 | ind_2 | 0 | 0 | 1 | 1 | 0.15 | 1.3 | 0.96 |
| fam_3 | ind_3 | 0 | 0 | 1 | 2 | 1.23 | 0.04 | 1.11 |
| fam_4 | ind_4 | 0 | 0 | 2 | 2 | 0.35 | 1.95 | NA |

- example.map: see plink-formatted map file above.
- fcGENE commands:
  - To create this format

*--oformat recodeA-dose*

**PLINK -formatted raw file (created with --recodeAD in plink ): example.raw**

- example.raw:

| FID | IID | PAT | MAT | SEX | PHENOTYPE | snp1_T | snp1_HET | snp2_C | snp2_HET |
| --- | --- | --- | --- | --- | --- | --- | --- | --- | --- |
| fam_1 | ind_1 | 0 | 0 | 1 | 1 | 2 | 0 | 0 | 0 |
| fam_2 | ind_2 | 0 | 0 | 1 | 1 | 0 | 0 | 1 | 1 |
| fam_3 | ind_3 | 0 | 0 | 1 | 2 | 1 | 1 | 0 | 0 |
| fam_4 | ind_4 | 0 | 0 | 2 | 2 | 0 | 0 | NA | NA |

- example.map: see plink formatted map file above.
- example_snpinfo.txt : Given previously.
- fcGENE command:

*./fcgene --recodeAD example.raw --map example.map --snpinfo example_snpinfo.txt\*

*--pedinfo example_pedinfo* (optional)

**PLINK -formatted covariate file: example.cov.txt**

- example.cov.txt

| FID | IID | pheno1 | pheno2 | covar_A | covar_B |
| --- | --- | --- | --- | --- | --- |
| fam_1 | ind_1 | 0 | 0 | 0 | 0.61 |
| fam_2 | ind_2 | 0 | 0 | 0 | 0.44 |
| fam_3 | ind_3 | 0 | 0 | 0 | 0.39 |
| fam_4 | ind_4 | 1 | 0 | 0 | 0.29 |

- fcGENE command: fcGENE can read this file and convert it into SNPTEST format using the following options:

*--covar example_plink_covariate.txt\*

*--covar-name pheno1,pheno2,covar_A,covar_B\*

*--covar-type P,B,D,C*

Here, command option *--covar* reads plink-formatted covariate file. Similarly, using command option *--covar-name*, one can determine the name of covariates. Command option *--covar-type* helps to determine the type of covariates. The letter *P* stands for binary phenotypes taking values of 0 and 1 respectively, *B* stands for Binary, *D* and *C* represent discrete and continues variables.

**Remarks**: More information on plink-formatted data can be found at

<http://pngu.mgh.harvard.edu/~purcell/plink/dataman.shtml>

**MaCH (Merlin) format files: example.ped and example.dat**

- example.ped

| fam_1 | ind_1 | 0 | 0 | m | T | T | T | T | G | G |
| --- | --- | --- | --- | --- | --- | --- | --- | --- | --- | --- |
| fam_2 | ind_2 | 0 | 0 | m | G | G | C | T | G | A |
| fam_3 | ind_3 | 0 | 0 | m | T | G | T | T | A | G |
| fam_4 | ind_4 | 0 | 0 | f | G | G | C | C | 0 | 0 |

or

| fam_1 | ind_1 | 0 | 0 | m | T/T | T/T | G/G |
| --- | --- | --- | --- | --- | --- | --- | --- |
| fam_2 | ind_2 | 0 | 0 | m | G/G | C/T | G/A |
| fam_3 | ind_3 | 0 | 0 | m | T/G | T/T | A/G |
| fam_4 | ind_4 | 0 | 0 | f | G/G | C/C | 0/0 |
|  |  |  |  |  |  |  |  |

- example.dat

| M | Snp1 |
| --- | --- |
| M | snp2 |
| M | snp3 |

- fcGENE commands:
  - To read files:

*./fcgene --ped example.ped --dat example.map*

- - To create files:

*--oformat mach*

**MaCH-formatted references: mach_ref.snps, mach_ref.hap**

- mach_ref.snps

| Snp1 |
| --- |
| Snp2 |
| Snp3 |

- mach_ref.hap

| fam_1->ind_1 | HAPLO1 | T | T | G |
| --- | --- | --- | --- | --- |
| fam_1->ind_1 | HAPLO2 | G | T | G |
| fam_2->ind_2 | HAPLO1 | G | T | A |
| fam_2->ind_2 | HAPLO2 | G | C | G |
| fam_3->ind_3 | HAPLO1 | T | T | G |
| fam_3->ind_3 | HAPLO2 | G | T | A |
| fam_4->ind_4 | HAPLO1 | T | C | A |
| fam_4->ind_4 | HAPLO2 | T | T | G |

- fcGENE commands:
  - To read files:

*./fcgene --mach-hap mach_ref.hap --mach-snp mach_ref.snp\*

*--force pheno=unaff,sex=m* (optional)

- - To create files: not applicable

**MaCH imputation outputs:**

- example.geno and example.info files:
  - example.geno

| fam_1->ind_1 | GENO | T/T | T/T | G/G |
| --- | --- | --- | --- | --- |
| fam_2->ind_2 | GENO | G/G | T/C | G/A |
| fam_3->ind_3 | GENO | T/G | T/T | G/A |
| fam_4->ind_4 | GENO | G/G | C/C | G/G |

- - example.info

| SNP | Al1 | Al2 | Freq1 | MAF | Quality | Rsq |
| --- | --- | --- | --- | --- | --- | --- |
| snp1 | T | G | 0.375 | 0.375 | 1 | 1 |
| snp2 | T | C | 0.625 | 0.375 | 1 | 1 |
| snp3 | G | A | 0.6825 | 0.3175 | 0.89 | 0.6022 |

- - fcGENE commands:
    - To read files:

*./fcgene --geno example.geno -- info example.info*

- - - To create files: not applicable
- example.mlgeno and example.mlinfo files:
  - example.mlgeno

| fam_1->ind_1 | ML_GENO | G/G | C/C | G/G |
| --- | --- | --- | --- | --- |
| fam_2->ind_2 | ML_GENO | T/T | T/C | G/A |
| fam_3->ind_3 | ML_GENO | T/G | C/C | G/A |
| fam_4->ind_4 | ML_GENO | T/T | T/T | G/G |

| SNP | Al1 | Al2 | Freq1 | MAF | Quality | Rsq |
| --- | --- | --- | --- | --- | --- | --- |
| snp1 | T | G | 0.4901 | 0.4901 | 0.7088 | 0.6784 |
| snp2 | T | C | 0.4596 | 0.4596 | 0.8106 | 0.7682 |
| snp3 | G | A | 0.6686 | 0.3314 | 0.6021 | 0.0853 |
|  |  |  |  |  |  |  |

- - example.mlinfo
  - fcGENE commands:
    - To read files:

*./fcgene --mach-mlgeno example.geno --mach-mlinfo example.info*

*--rsq 0.3* (optional) *--maf-thresh 0.1* (optional)

- - - To create files with fcGENE: not applicable
- example.mlprob and example.mlinfo files:

| fam_1->ind_1 | ML_PROB | 0.006 | 0.14 | 0.081 | 0.407 | 0.365 | 0.478 |
| --- | --- | --- | --- | --- | --- | --- | --- |
| fam_2->ind_2 | ML_PROB | 0.301 | 0.516 | 0.393 | 0.535 | 0.395 | 0.5 |
| fam_3->ind_3 | ML_PROB | 0.05 | 0.364 | 0.065 | 0.397 | 0.271 | 0.544 |
| fam_4->ind_4 | ML_PROB | 0.383 | 0.472 | 0.886 | 0.11 | 0.519 | 0.403 |

- - example.mlprob
  - **exa**
  - example.mlinfo (see above)
  - fcGENE commands:
    - To read files:

*./fcgene –mach-mlprob example.geno -- mach-mlinfo example.info\*

*--rsq 0.3* (optional) *--maf-thresh 0.1* (optional)

*--pedinfo example_pedinfo.txt --snpinfo example_snpinfo.txt* (optional)

- - - To create files: not applicable

**MINIMAC format :**

- example.ped (see mach-formatted ped file )
- example.snps (see mach-formatted ref.snps file )
- fcGENE commands:
  - To read files : *./fcgene --ped example.ped -- snps example.snps*
  - To create files: *--oformat minimac*

**MINIMAC imputation outputs: see mach imputation outputs**

**IMPUTE (CHIAMO) format files:**

- example.gens

| snp1 | Snp1 | 16212142.00 | T | G | 0.01 | 0.14 | 0.85 | 0.30 | 0.52 | 0.18 | 0.05 | 0.36 | 0.59 | 0.38 | 0.47 | 0.15 |
| --- | --- | --- | --- | --- | --- | --- | --- | --- | --- | --- | --- | --- | --- | --- | --- | --- |
| snp2 | Snp2 | 20278224.00 | T | C | 0.08 | 0.41 | 0.51 | 0.39 | 0.54 | 0.07 | 0.07 | 0.40 | 0.54 | 0.89 | 0.11 | 0.00 |
| snp3 | Snp3 | 20304703.00 | G | A | 0.37 | 0.48 | 0.16 | 0.40 | 0.50 | 0.11 | 0.27 | 0.54 | 0.19 | 0.52 | 0.40 | 0.08 |

- example.strand

| rs1 | + |
| --- | --- |
| rs2 | + |
| rs3 | + |

- fcGENE commands:
  - To read files:

*--gens example.gens --strand example.strand\*

*--thresh maxProb --pedinfo example_pedinfo.txt* (optional)

- - To create files: *--oformat impute*

**IMPUTE reference files:**

- Impute_ref.hap

| 0 | 0 | 0 | 1 | 1 | 0 | 1 | 1 |
| --- | --- | --- | --- | --- | --- | --- | --- |
| 1 | 1 | 0 | 0 | 1 | 1 | 0 | 1 |
| 1 | 1 | 0 | 0 | 0 | 1 | 1 | 0 |

This file contains 3 SNPs and four individuals. Each row represents a SNP. Each two columns represent an individual.

- Impute_genetic_map.txt

| position | CEU_rate(cM/Mb) | Genetic_Map(cM) |
| --- | --- | --- |
| 14431347 | 12.83039317 | 0 |
| 14432618 | 12.87908888 | 0.01630743 |
| 14433624 | 12.87908888 | 0.029263793 |
| 14433659 | 12.87568372 | 0.029714561 |
| 14433758 | 12.85861428 | 0.030989254 |
| 14434713 | 12.60322816 | 0.043269231 |
| 14435070 | 5.89938528 | 0.047768583 |
| 14439734 | 1.375554222 | 0.0752833160 |
|  |  |  |

- Impute_ref.legend

| rsID | position | a0 | a1 |
| --- | --- | --- | --- |
| rs7288834 | 16212142 | G | T |
| rs16978746 | 20278224 | T | C |
| rs5754387 | 20304703 | G | A |

- fcGENE commands:
  - To read impute references with fcGENE:

*./fcgene --impute-hap impute_ref.hap --impute-legend impute_ref.legend\*

*--force pheno=unaff,sex=m* (optional)

- - To create files: not applicable

**IMPUTE outputs:**

- example_impute2:

| snp1 | snp1 | 16212142.00 | T | G | 0.06 | 0.35 | 0.59 | 0.01 | 0.13 | 0.85 | 0.06 | 0.35 | 0.59 | 0.04 | 0.31 | 0.66 |
| --- | --- | --- | --- | --- | --- | --- | --- | --- | --- | --- | --- | --- | --- | --- | --- | --- |
| snp2 | snp2 | 20278224.00 | T | C | 0.25 | 0.50 | 0.25 | 0.03 | 0.55 | 0.41 | 0.07 | 0.77 | 0.16 | 0.16 | 0.45 | 0.39 |
| snp3 | snp3 | 20304703.00 | G | A | 0.15 | 0.46 | 0.39 | 0.38 | 0.58 | 0.04 | 0.10 | 0.29 | 0.61 | 0.72 | 0.23 | 0.06 |

- example_info

| snp_id | rs_id | position | exp_freq_a1 | info | certainty | type | Concord_type2 | r2_type2 |
| --- | --- | --- | --- | --- | --- | --- | --- | --- |
| snp1 | snp1 | 16212142 | 0.815 | -0.01 | 0.673 | 3 | -1 | -1 |
| snp2 | snp2 | 20278224 | 0.588 | 0.214 | 0.567 | 3 | -1 | -1 |
| snp3 | snp3 | 20304703 | 0.469 | 0.211 | 0.592 | 3 | -1 | -1 |

- fcGENE commands:
  - To read files:

*./fcgene --gens example.impute2 --info example.impute2_info\*

*--thresh 0.9* (optional) *--info-thresh 0.3\* (optional)

*--maf-thresh 0.1\* (optional)

*-- pedinfo example_pedinfo.txt* (optional)

- - To create files: not applicable

**BEAGLE input files:**

- example.bgl

| I | id | ind_1 | ind_1 | ind_2 | ind_2 | ind_3 | ind_3 | Ind_4 | ind_4 |
| --- | --- | --- | --- | --- | --- | --- | --- | --- | --- |
| A | phenotype | 1 | 1 | 1 | 1 | 2 | 2 | 2 | 2 |
| M | snp1 | G | G | T | G | G | G | T | G |
| M | snp2 | C | C | T | C | C | C | T | T |
| M | snp3 | G | A | G | A | G | A | G | G |

- fcGENE commands:
  - To read files: *./fcgene –bgl example.bgl*
  - To create files: *--oformat beagle*

**BEAGLE references file:**

- beagle_ref.bgl : see example_beagle.bgl file
- fcGENE commands:
  - To read data:

*./fcgene --bgl beagle_ref.bgl\*

*--force pheno=unaff,sex=m* (optional)

- - To create files: not applicable

**BEAGLE-imputation output files**

- example.gprobs:

| marker | alleleA | alleleB | ind_1 | ind_1 | ind_1 | ind_2 | ind_2 | Ind_2 | ind_3 | ind_3 | Ind_3 | ind_4 | ind_4 | ind_4 |
| --- | --- | --- | --- | --- | --- | --- | --- | --- | --- | --- | --- | --- | --- | --- |
| Snp1 | G | T | 1 | 0 | 0 | 0 | 1 | 0 | 1 | 0 | 0 | 0 | 1 | 0 |
| Snp2 | C | T | 1 | 0 | 0 | 0 | 1 | 0 | 1 | 0 | 0 | 0 | 0 | 1 |
| Snp3 | G | A | 0 | 1 | 0 | 0 | 1 | 0 | 0 | 1 | 0 | 1 | 0 | 0 |

- example.bgl.r2

| Snp1 | 1.000 |
| --- | --- |
| Snp2 | 1.000 |
| Snp3 | 1.000 |

- fcGENE commands:
  - To read files:

*./fcgene --bgl-gprobs example.bgl.gprobs\*

*--bgl-rsq example.bgl.r2 --rsq-thresh 0.3\* (optional)

*--pedinfo example_pedinfo.txt --snpinfo example_snpinfo.txt* (optional)

- - To create files: not applicable

**BIMBAM input files:**

- example.geno

| 4 |  |  |  |  |
| --- | --- | --- | --- | --- |
| 3 |  |  |  |  |
| IND, | ind_1, | ind_2, | ind_3, | ind_4 |
| snp1, | TG, | GG, | TG, | GG |
| snp2, | TT, | CT, | TT, | CC |
| snp3, | GG, | GA, | AG, | ?? |

- example.pheno

| 1 |
| --- |
| 1 |
| 0 |
| 0 |

| snp1, | 16212142 |
| --- | --- |
| snp2, | 20278224 |
| snp3, | 20304703 |

- example.pos.txt
- fcGENE commands:
  - To read files: *./fcgene -- example.geno.txt --pos example.pos.txt*
  - To create files: *--oformat bimbam*

**BIMBAM imputation output-files:**

- example. mean.genotype.txt

| snp1 | T | G | 2 | 0 | 1 | 0 |
| --- | --- | --- | --- | --- | --- | --- |
| snp2 | C | T | 0 | 1 | 0 | 2 |
| snp3 | A | G | 0 | 1 | 1 | 0.751 |

- example.genotype.distribution.txt

| snp1 | T | G | 0 | 0 | 1 | 0 | 0 | 1 | 1 | 0 |
| --- | --- | --- | --- | --- | --- | --- | --- | --- | --- | --- |
| snp2 | C | T | 1 | 0 | 0 | 1 | 1 | 0 | 0 | 0 |
| snp3 | A | G | 1 | 0 | 0 | 1 | 0 | 1 | 0.627 | 0.075 |

- Example.best.guess.genotype.txt

| Snp1 | GT | GG | GT | GG |
| --- | --- | --- | --- | --- |
| Snp2 | TT | TC | TT | CC |
| Snp3 | GG | GA | GA | GG |

| ## af is the allele freq for A | | | | | |
| --- | --- | --- | --- | --- | --- |
| Rs | A | B | af | chr | Pos |
| snp1 | T | G | 0.25 | 22 | 16212142 |
| snp2 | C | T | 0 | 22 | 20278224 |
| snp3 | A | G | 0 | 22 | 20304703 |

- Example.snpinfo.txt

- fcGENE command:
  - To read files:

*./fcgene --wbg example.best.guess.genotype.txt --pos example.snpdata.txt*

*--maf-thresh 0.1* (optional)

*./fcgene --wgd example.genotype.distribution.txt --pos example.snpdata.txt\*

*--maf-thresh 0.1* (optional)

- - To create files: not applicable

**Haploview linkage format**

- Example.ped

| fam_1 | ind_1 | 0 | 0 | 1 | 1 | T | T | T | T | G | G |
| --- | --- | --- | --- | --- | --- | --- | --- | --- | --- | --- | --- |
| fam_2 | ind_2 | 0 | 0 | 1 | 1 | G | G | C | T | G | A |
| fam_3 | ind_3 | 0 | 0 | 1 | 2 | T | G | T | T | A | G |
| fam_4 | ind_4 | 0 | 0 | 2 | 2 | G | G | C | C | 0 | 0 |

- Example.info

| Snp1 | 16212142 |
| --- | --- |
| Snp2 | 20278224 |
| Snp3 | 20304703 |

- fcGENE commands:
  - To read files: not applicable
  - To create files: *--oformat haploview*

**Eigensoft input format**

- example.ped

| fam_1 | ind_1 | 0 | 0 | 1 | 1 | T | T | T | T | G | G |
| --- | --- | --- | --- | --- | --- | --- | --- | --- | --- | --- | --- |
| fam_2 | ind_2 | 0 | 0 | 1 | 1 | G | G | C | T | G | A |
| fam_3 | ind_3 | 0 | 0 | 1 | 2 | T | G | T | T | A | G |
| fam_4 | ind_4 | 0 | 0 | 2 | 2 | G | G | C | C | 0 | 0 |

- Example.pedind

| Fam_1 | ind_1 | 0 | 0 | 1 | 1 |
| --- | --- | --- | --- | --- | --- |
| Fam_2 | ind_2 | 0 | 0 | 1 | 1 |
| Fam_3 | ind_3 | 0 | 0 | 1 | 2 |
| Fam_4 | ind_4 | 0 | 0 | 2 | 2 |

- Example.pedsnp

| 22 | snp1 | 0.02 | 16212142 | T | G |
| --- | --- | --- | --- | --- | --- |
| 22 | snp2 | 0.13 | 20278224 | C | T |
| 22 | snp3 | 0.13 | 20304703 | A | G |
|  |  |  |  |  |  |

- fcGENE commands:
  - To read files: not applicable
  - To create files with fcGENE: *--oformat eigensoft*

**SNPTEST format**

- example.gen : see impute-formatted example.gens
- example.strand : see impute-formatted example.strand.txt
- example.sample :

| ID_1 | ID_2 | missing | sex | status |
| --- | --- | --- | --- | --- |
| 0 | 0 | 0 | B | B |
| fam_1 | ind_1 | 0 | 1 | 0 |
| fam_2 | ind_2 | 0 | 1 | 0 |
| fam_3 | ind_3 | 0 | 1 | 1 |
| fam_4 | ind_4 | 0 | 0 | 1 |

- fcGENE commands:
  - To read files: not applicable
  - To create files with fcGENE:

*--oformat snptest\*

*--covar example/plink_cov.txtn\*

*--covar-name pheno1,pheno2,covar_A,covar_B\*

*--covar-type P,B,D,C*

**Standard text files saved as minor allele coding (012):**

- Example_genotype.txt

| SMAPLE_ID | snp1 | Snp2 | snp3 |
| --- | --- | --- | --- |
| ind_1 | 2 | 0 | 0 |
| ind_2 | 0 | 1 | 1 |
| ind_3 | 1 | 0 | 1 |
| ind_4 | 0 | 2 | NA |

In this genotype file, ‘0’ means homozygote major of alternative allele,’1’ denotes the heterozygote and ‘2’ denotes the homozygote major of reference allele (see below example_alleleInfo.txt)

- example_affection.txt

| SMAPLE_ID | AFFSTAT |
| --- | --- |
| ind_1 | 0 |
| ind_2 | 0 |
| ind_3 | 1 |
| ind_4 | 1 |

- example_alelleinfo.txt (Allele1 is the reference allele and allele2 is alternative allele)
  nchr rsid allele1 allele2

22 snp1 T G

22 snp2 C T

22 snp3 A G

- example.freq: (A1 is minor allele )

| CHR | SNP | A1 | A2 | MAF | NCHROBS |
| --- | --- | --- | --- | --- | --- |
| 22 | snp1 | G | T | 0.375 | 8 |
| 22 | snp2 | C | T | 0.375 | 8 |
| 22 | snp3 | A | G | 0.25 | 6 |

- fcGENE commands:
  - To write files:

*--oformat r* (or *--oformat R*)

- - To read the above mentioned genotype formats:

./fcgene --rgeno genotype data --snpinfo allele_info.txt

- - To transpose genotype data:

./fcgene --rgeno genotype data --snpinfo allele_info.txt --transpose --oformat r

**Standard text files with genotype coding as expected dose of minor alleles:**

- Example_genotype.txt

| SMAPLE_ID | snp1 | Snp2 | snp3 |
| --- | --- | --- | --- |
| ind_1 | 1.89 | 0.19 | 0.18 |
| ind_2 | 0.15 | 1.3 | 0.96 |
| ind_3 | 1.23 | 0.04 | 1.11 |
| ind_4 | 0.35 | 1.95 | NA |

- fcGENE commands: *--oformat r-dose* (or *--oformat R-dose*)
- example_affection.txt : see previous type

**fcGENE’s pedInfo and SNPInfo files**:

- example_pedinfo.txt

| Famid | indid | matid | patid | Sex | phenotype |
| --- | --- | --- | --- | --- | --- |
| Fam_1 | ind_1 | 0 | 0 | 1 | 1 |
| Fam_2 | ind_2 | 0 | 0 | 1 | 1 |
| Fam_3 | ind_3 | 0 | 0 | 1 | 2 |
| Fam_4 | ind_4 | 0 | 0 | 2 | 2 |

- fcGENE’s commands:
  - To read files: *--pedinfo example_pedinfo.txt*
- example_snpinfo.txt

| Nchr | snpid | rsid | bp | cm_pos | allele1 | Allele2 |
| --- | --- | --- | --- | --- | --- | --- |
| 22 | snp1 | snp1 | 16212142 | 0.02 | T | G |
| 22 | snp2 | snp2 | 20278224 | 0.13 | T | C |
| 22 | snp3 | snp3 | 20304703 | 0.13 | G | A |

- fcGENE commands:
  - To read file: *--snpinfo example_pedinfo.txt*
  - *--snpinfo* and *--pedinfo* commands can be used with any type of data format readable by fcGENE. An example is given below:

*./fcgene --wgd example.genotype.distribution.txt --pos example.snpdata.txt\*

*--maf-thresh 0.1 --snpinfo example_snpinfo.txt –pedinfo example_pedinfo.txt*

**Commands for quality control:**

In this section, we present fcGENE commands required for quality control prior to or after genotype imputation.

**Calculation of SNP-wise call rate and individual wise call rate:** In order to calculate SNP-wise and individual-wise call rate, one can use command option *--crate*. This command will produce two files, namely “snp_crate.txt” and “indiv_crate.txt”.

- example_snp_crate.txt:

| CHR | SNP | CRATE |
| --- | --- | --- |
| 22 | snp1 | 1 |
| 22 | snp2 | 1 |
| 22 | snp3 | 0.75 |
|  |  |  |

- example_indiv_crate.txt:

| FID | IID | CRATE |
| --- | --- | --- |
| fam_1 | ind_1 | 1 |
| fam_2 | ind_2 | 1 |
| fam_3 | ind_3 | 1 |
| fam_4 | ind_4 | 0.75 |

- Example of fcGENE command: *./fcgene --bgl-gprobs example.bgl.gprobs --crate*

**Calculation of p-Values of HWE:** This option performs asymptotic Chi-square testing of Hardy-Weinberg equilibrium. For more details see Wigginton JE, Cutler DJ and Abecasis GR.

“A Note on Exact Tests of Hardy-Weinberg Equilibrium”, Am J Hum Genet (2005) 76: 887-93.

- Resulting file: snp_hwe.txt:

| CHR | SNP | PVALUE_EXACT |
| --- | --- | --- |
| 22 | snp1 | 0.428571 |
| 22 | snp2 | 0.428571 |
| 22 | snp3 | 1 |

- fcGENE commands:
  - To calculate p-values for testing Hardy-Weinberg Disequilibrium for each SNP, use command option *--hardy*.
  - Example of fcGENE command:

*./fcgene --ped example.ped --map example.map --hardy*

**Calculation of allele frequencies**

- example_snp.frq:

| CHR | SNP | A1 | A2 | MAF | NCHROBS |
| --- | --- | --- | --- | --- | --- |
| 22 | snp1 | G | T | 0.375 | 8 |
| 22 | snp2 | C | T | 0.375 | 8 |
| 22 | snp3 | A | G | 0.25 | 6 |

- fcGENE commands: Calculation of minor allele frequencies can be performed with command *--freq*, for example *./fcgene --ped example.ped --map example.map --freq*

**Remark:** Calculation of call rate, p-values of HWE, and MAF can be performed within a single command:

*./fcgene --ped example.ped --map example.map\*

*--freq --crate –hardy --out example*

**Discarding SNPs from analysis:** The following command first excludes SNPs specified in the file *snplist.txt* and then performs other tasks such as converting format into BIMBAM or calculating HWE and MAF.

*./fcgene --ped example.ped --map example.map\*

*--exclude snplist.txt --hardy --maf --oformat bimbam\*

*--out bimbam/plink_bimbam*

**Discarding samples from analysis:**  Using command option *--remove*, one can discard individuals from subsequent analyses. The following command first excludes individuals specified in the file *indivlist.txt* and then performs other tasks such as converting format into IMPUTE format and calculating call rates.

*./fcgene --ped example.ped --map example.map\*

*--remove indivlist.txt --crate --oformat impute\*

*--out impute/plink_impute*

**Quality control of SNPs and Individuals:** In order to filter SNPs and individuals according to specified quality criteria (SNP-wise call rate, HWE and MAF, sample-wise call rate), we can use command options *--filter-snp* and *--filter-indiv*. The following command filters SNPs and individuals first, and then converts the genotype data into IMPUTE format

*./fcgene --ped example.ped --map example.map\*

*--filter-snp hwe=1e-2,maf=0.1,crate=0.95 --filter-indiv crate=0.95*

*--oformat impute\*

*--out impute/plink_impute*

**Splitting genotype data on the basis of SNPs and Individuals**: fcGENE can split a genotype data into any number of subsets. Moreover, the genotype data can have any format mentioned previously and splitting can be performed SNP-wise and sample-wise. While performing sample-wise splitting one can provide lower and upper indices of samples to be extracted using command option --isplit as follows:

*./fcgene --file example --isplit 1-10,5-20,9-30 --out split_data*

Different ranges of indices of samples to be extracted are separated by a comma. Hence, this example command will split example.ped and example.map into three datasets, where the first dataset contains the first ten samples of the sample list, the second dataset contains samples with index between 5 and 20 and the third data set contains samples with index between 9 and 30.

To split data on the basis of SNPs, one can use two commands namely --ssplit and --bpsplit commands. “--ssplit” requires the lower and the upper index of SNPs to be extracted. Similarly “--bpslit” requires upper and lower limits of base pair positions of SNPs to be extracted. This option is useful if considering genotype data of a single chromosome. Again different ranges can be extracted if separated by a comma. Examples of SNP-wise splitting are given below:

*./fcgene --mfile example --ssplit 1-100,50-200,100-1000 --out split_data*

*./fcgene --mfile example --bpsplit 16212142-24236898, 24540850- 24540850 --out split_data*

One can arbitrarily combine SNP-wise and sample-wise splitting to define different subsets of genotype data.

*./fcgene --mfile example --isplit 1-100,50-200\*

*--bpsplit 16212142-24236898, 24540850- 24540850 –out split_data*

*./fcgene --mfile example --isplit 1-100, --ssplit 1-500, 501-1000 –out split_data*

**Creation of new sample ids for family data**: This option is used to construct hybrid IDs on the basis of family data. We can specify an adequate rule using command option “--iid” in the following way:

| Command option | New IDs |
| --- | --- |
| - -iid famid,iid,sep=_ | famid_iid |
| - -iid famid,iid,patid,matid,sep=_ | famid_iid_patid_matid |
| - -iid famid,iid,patid,matid,sep=- | famid-iid-patid-matid |
| - -iid famid,iid,patid,matid | famidiidpatidmatid |

**Example:** *./fcgene --example.ped --map example.map --iid fid,iid,sep=_ --oformat haploview*

Here, command option *“-- iid fid,iid,sep= _”* creates new dummy sample IDs of the form “FamilyID_IndividualID”.

**Multiple commands at a time:**

For the efficient use of fcGENE, one can apply multiple commands at a time. If we want to perform two or more tasks in fcGENE, these tasks can be separated by command options *--new-start* and *--new-end*. The following examples show how to perform multiple tasks in fcGENE simultaneously.

- When a fcGENE command contains multiple format converting tasks, each new task, except for the first, is separated by command identifiers *--new-start* and *--new-end*. The following command reads two PLINK-formatted files, and convert the first into MaCH and second into IMPUTE format.

*./fcgene --ped example1.ped --map example1.map\*

*--oformat mach --out mach/example\*

*--new-start\*

*--dosage example2.dose --fam example2.fam\*

*--map example2.map --oformat impute --out impute/example*

*--new-end*

- In the next example, two datasets were merged and then converted into EIGENSOFT format

*./fcgene --ped example1.ped --map example1.map\*

*--new-start\*

*--gens impute/example2.gens --pedinfo impute/example2_pedinfo.txt \*

*--merge\*

*--new-end \*

*--out eigensoft/example_merge --oformat eigensoft*

- The following command reads three datasets, merge the first two of them before the merged datasets are converted into PLINK dosage format. Call rate, HWE and MAFs are calculated for the third dataset. Then it is converted into BEAGLE format.
  - *./fcgene --ped example1.ped --map example1.map\*

*--new-start\*

*--ped mach/example2.ped --dat mach/example2.dat\*

*--snpinfo mach/example2_snpinfo --filter-snp hwe=1e-2\*

*--merge\*

*--new-end\*

*--new-start \*

*--gens impute/example3.gens --pedinfo impute/example3_pedinfo.txt \*

*--hardy --crate --freq --oformat beagle \*

*--out beagle/impute_beagle\*

*--new-end \*

*--out plink/example_dosage --oformat plink-dosage*
